# Comparison between oblique lumbar interbody fusion and posterior lumbar interbody fusion for the treatment of lumbar degenerative diseases: a systematic review and meta-analysis

**DOI:** 10.1186/s13018-023-04312-4

**Published:** 2023-11-10

**Authors:** Bochen An, Bowen Ren, Zhenchuan Han, Keya Mao, Jianheng Liu

**Affiliations:** 1 Medical School of Chinese PLA, Beijing, 100089 China; 2 Department of Orthopedics, Chinese PLA General Hospital, Beijing, 100089 China; 3 Department of Orthopedics, Chinese PLA Rocket Force Characteristic Medical Center, Beijing, 100088 China

**Keywords:** Lumbar degenerative diseases, Lumbar fusion, Meta-analysis, Oblique lateral approach vertebral fusion, Posterior approach vertebral fusion, Meta-analysis, Systematic review

## Abstract

**Background:**

Although oblique lumbar interbody fusion (OLIF) has produced good results for lumbar degenerative diseases (LDDs), its efficacy vis-a-vis posterior lumbar interbody fusion (PLIF) remains controversial. This meta-analysis aimed to compare the clinical efficacy of OLIF and PLIF for the treatment of LDDs.

**Methods:**

A comprehensive assessment of the literature was conducted, and the quality of retrieved studies was assessed using the Newcastle–Ottawa Scale. Clinical parameters included the visual analog scale (VAS), and Oswestry Disability Index (ODI) for pain, disability, and functional levels. Statistical analysis related to operative time, intraoperative bleeding, length of hospital stay, lumbar lordosis angle, postoperative disc height, and complication rates was performed. The PROSPERO number for the present systematic review is CRD42023406695.

**Results:**

In total, 574 patients (287 for OLIF, 287 for PLIF) from eight studies were included. The combined mean postoperative difference in ODI and lumbar VAS scores was − 1.22 and − 0.43, respectively. In postoperative disc, height between OLIF and PLIF was 2.05. The combined advantage ratio of the total surgical complication rate and the mean difference in lumbar lordosis angle between OLIF and PLIF were 0.46 and 1.72, respectively. The combined mean difference in intraoperative blood loss and postoperative hospital stay between OLIF and PLIF was − 128.67 and − 2.32, respectively.

**Conclusion:**

Both the OLIF and PLIF interventions showed good clinical efficacy for LDDs. However, OLIF demonstrated a superior advantage in terms of intraoperative bleeding, hospital stay, degree of postoperative disc height recovery, and postoperative complication rate.

## Background

Lumbar degenerative diseases (LDDs) are the leading cause of chronic lower back pain in older adults worldwide [1–3], and their prevalence continuously increases as the proportion of the elderly increases in the global population [4]. Spinal fusion is the gold standard for the treatment of LDDs in patients for whom conservative treatment fails and whose symptoms worsen. In 1952, Cloward et al. [5] reported posterior lumbar interbody fusion (PLIF), a surgical method that uses a posterior approach to the lumbar spine to complete the target disc exposure by stripping the muscle tissue on both sides of the spinous process, cutting out parts of the lamina and ligamentum flavum, and exposing the dural sac and nerve roots [6]. The advantages of this procedure include a wide surgical field of view and adequate exposure of the nerve root, without affecting the blood supply to the graft through a posterior exposure [7]. In addition, it allows for a potential 360° fusion through a single incision.

However, PLIF has some disadvantages such as long surgical incisions, paravertebral medical injuries associated with prolonged muscle contraction, and a high incidence of degenerative lesions in adjacent segments of the spine [8, 9]. In 1997, Mayer et al. [10] proposed an approach involving the abdominal lumbar major and vascular sheath gap for the treatment of degenerative lumbar spine diseases. In 2012, Silvestre et al. [11, 12] reported on oblique lumbar interbody fusion (OLIF). Distinct from the traditional PLIF procedure, OLIF utilizes the anatomical space between the psoas major muscle and the large abdominal vascular sheath, without detaching the psoas major muscle. This approach effectively avoids damage to the blood vessels and lumbar plexus nerves caused by direct lateral interbody fusion to separate the lumbar major muscle [13]. However, complications such as transient thigh numbness and hip flexion weakness may occur following OLIF [14].

Currently, both surgical approaches have achieved good results for the treatment of LDDs. However, there is a lack of evidence on the superiority of OLIF over PLIF. Therefore, this meta-analysis aimed to evaluate and compare the effectiveness of OLIF and PLIF for the treatment of LDDs in terms of clinical efficacy, perioperative surgical indicators, and complication rates. This systematic review provides a reference for clinicians in selecting surgical procedures for the treatment of LDDs.

## Methods

### Search strategy

A systematic literature review was conducted using the Preferred Reporting Items for Systematic Evaluations and Meta-Analyses (PRISMA) guidelines [15]. We retrieved retrospective case–control studies that compared OLIF and PLIF for the treatment of LDDs. PubMed, MEDLINE, Cochrane, Web of Science, China National Knowledge Infrastructure, and Offshore Vessel Inspection Database repositories were searched from the time of their inception until April 2023. To optimize search sensitivity, the following keyword combinations were used: “OLIF” or “oblique lumbar interbody fusion” and “PLIF” or “posterior lumbar interbody fusion.”

We also identified relevant studies from the references cited in the retrieved studies; two researchers (A.-B.C. and R.-B.W.) independently evaluated the titles and abstracts of all search results and further evaluated those that appeared relevant. Any differences in opinion were resolved through discussion with a third party (H.-Z.C.). Our meta-analysis review protocols were registered on PROSPERO (International Prospective Register of Systematic Reviews; Registration Number CRD42023406695).

### Inclusion and exclusion criteria

The inclusion criteria were as follows: (1) all relevant clinical studies/original articles (randomized controlled trials [RCTs] or retrospective studies); (2) lumbar degenerative diseases encompassing conditions such as degenerative disc degeneration, spondylosis, lumbar spinal stenosis, and degenerative spondylolisthesis; (3) those comparing OLIF combined with percutaneous pedicle screw fixation and PLIF in humans; and (4) those reporting clinical, perioperative, or postoperative assessment metrics, such as the visual analog scale (VAS) [16] and/or the Oswestry Disability Index (ODI) [17] and complication rates. The exclusion criteria were as follows: (1) single-arm studies without control groups; (2) studies without relevant data; and (3) case reports, technical notes, reviews, duplicate studies, and conference reports.

### Literature screening

#### Quality evaluation

The quality of each study was independently assessed by two reviewers (A.-B.C. and R.-B.W.). Non-RCTs were assessed using the Newcastle–Ottawa Scale [18]. Selection, comparability, and exposure/outcome of each study were fully assessed, and those achieving more than 5 stars during the quality screening were included in the analysis.

#### Data extraction

Two reviewers (A.-B.C. and R.-B.W.) independently collected data using standard data extraction methods. The following general characteristics were extracted from each study: author, country, year, study design, number of cases, diagnosis, method of surgical intervention, patient sex, and duration of follow-up. Primary outcomes were VAS scores for lower back and leg pain, ODI scores for lower back pain, lumbar lordosis angle [19], and disc height [20]. All outcomes were measured preoperatively and postoperatively at follow-up (3–12 months). Secondary outcomes were perioperative parameters (operative time, intraoperative blood loss, length of hospital stay) and complication rates.

### Statistical analysis

Data were analyzed using the Review Manager software, version 5.4 (Cochrane Collaboration, Oxford, United Kingdom). Continuous data are presented as the mean differences (MDs) and 95% confidence intervals (CIs). Dichotomous variables in comparative studies were estimated using dominance (odds ratio [OR]) or risk ratios. Continuous variables were analyzed using standardized MDs or weighted MDs (WMDs).

Chi-squared (*χ*^2^) and *I*^2^ tests were used to assess heterogeneity. If *I*^2^ was ≥ 50% or *P* was < 0.1, statistical heterogeneity among studies was considered, and a random-effects model was used. Otherwise, if *I*^2^ was < 50% or *P* was > 0.1, no statistical heterogeneity between studies was considered, and a fixed-effects model was used. Statistical significance was set at *P* < 0.05. Forest plots were created to represent the results of the various types of studies and summary estimates of the effects.

## Results

### Inclusion of studies and quality evaluation

**Fig. 1:**
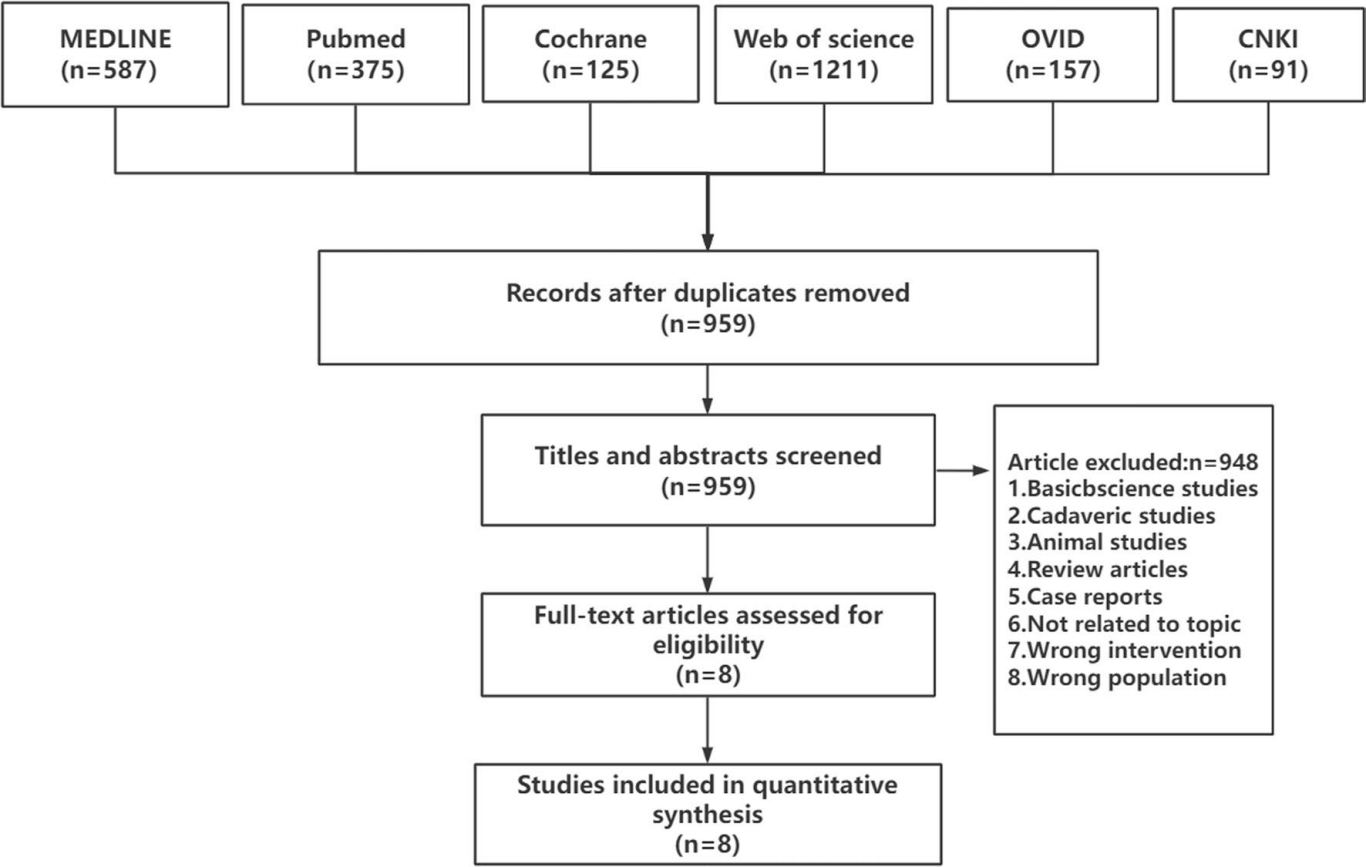
Schematic of selection process for meta-analysis

The PRISMA flowchart (Fig. 1) shows the literature search process. After removing 1587 duplicate studies, 959 studies from the initial screening were obtained from a search of six electronic databases. Eight studies that met the inclusion criteria were retained for analysis. All were retrospective case–control studies, demonstrating a moderate-to-high quality according to the Newcastle–Ottawa Scale (Table 1).

**Table 1:** Quality assessment of the included studies

Reference	Selection				Exposure				
	Is the case definition adequate?	Representativeness of the cases	Selection of controls	Definition of controls	Comparability of cases and controls based on the design or analysis	Ascertainment of exposure	Same method of ascertainment for cases and controls	Nonresponse rate	Total scores (of 9)
Cen et al. [24]	*	*		*	**	*	*		7
Li et al. [11]	*	*		*	**	*	*	*	8
Chen et al. [25]	*	*		*	**	*	*	*	8
Cho et al. [12]	*	*		*	**	*	*	*	8
Du et al. [27]	*	*		*	**	*	*	*	8
Kang et al. [28]	*	*		*	**	*	*	*	8
Li et al. [23]	*	*		*	**	*	*	*	8
Zhao et al. [26]	*	*		*	**	*	*	*	8

NOS uses the semiquantitative principle of star system to evaluate the quality of literature, with a full score of 9 stars

### Characteristics of the included studies

In total, 574 patients across eight studies were enrolled in this meta-analysis and were equally divided between the OLIF and PLIF groups (*n* = 287 each). The mean ages of the patients in the OLIF and PLIF groups were 60.03 and 59.42 years, respectively. Demographic data are summarized in Table 2.

**Table 2:** Characteristics of the included studies

Reference	Year	Country	Study design	Number of cases (OLIF/PLIF)	Age (years) (OLIF/PLIF)	Diagnosis of diseases	Follow-up (months) (OLIF/PLIF)	Closing indicators
Cen et al	2018	China	Retrospective	28/28	45.2/43.5	DLS	Mean 12	①③⑤⑥⑦
Li et al	2022	China	Retrospective	51/52	58.42 ± 2.63/57.90 ± 2.87	DLSS	Mean 12	①②⑥⑦⑧
Chen et al	2022	China	Retrospective	38/38	63.26 ± 6.31/64.42 ± 5.13	DLS	–	②③④⑤⑥⑦⑧
Cho et al	2021	Korea	Retrospective	28/31	69.7 ± 6.9/67.4 ± 7.6	DLS	OLIF(27.7 ± 21.7) PLIF(34.9 ± 22.6)	①②③④⑤⑥⑦⑨
Du et al	2023	China	Retrospective	24/30	61.38 ± 6.79/60.83 ± 6.67	DLS	Mean 6	①③④⑤⑥⑦⑧⑨
Kang et al	2022	Korea	Retrospective	46/42	65.04 ± 6.90/65.45 ± 7.95	LDDs	Mean 12	①②③⑤⑥⑦⑧
Li et al	2020	China	Retrospective	20/22	53.38 ± 4.19/53.62 ± 4.63	DLS	–	③④⑤⑥⑦⑨
Zhao et al	2021	China	Retrospective	52/44	63.47 ± 9.26/62.27 ± 9.08	LDDs	Mean 6	①③⑤⑥⑦

① VAS back pain ② VAS leg pain ③ Oswestry Disability Index ④ lumbar lordosis angle ⑤ Perioperative complications ⑥ Operative time ⑦ Blood loss

⑧ Hospital stay ⑨ Disc height

### Clinical results

**Fig. 2:**
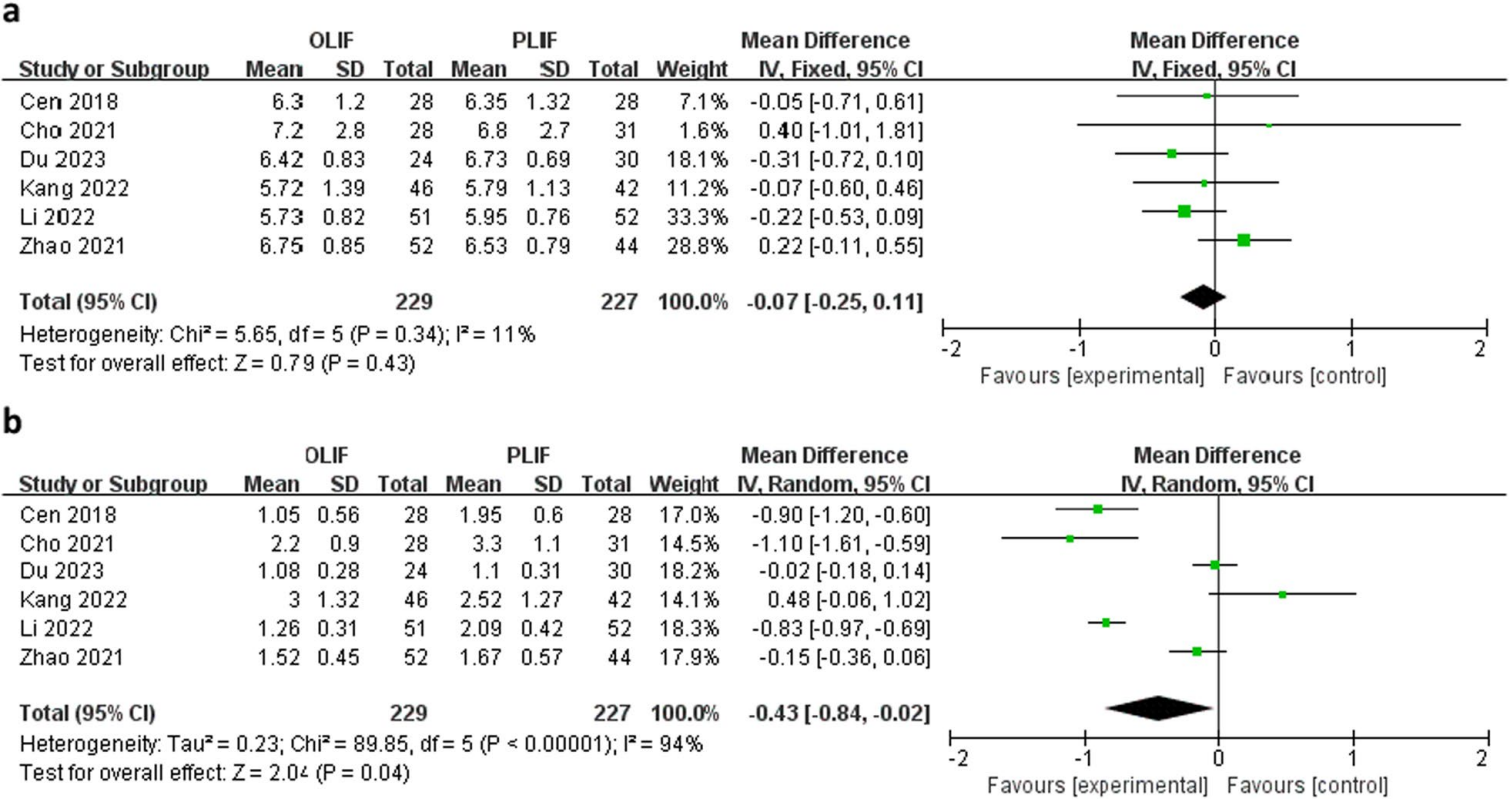
Forest plots of preoperative, **a** postoperative, **b** lumbar visual analog scale scores between OLIF and PLIF. *CI* confidence interval, *IV* inverse variance, *df* degrees of freedom, *SD* standard deviation

Six studies assessed lower back pain using the VAS. As shown in Fig. 2a, there was no significant difference in the mean preoperative VAS low back pain scores between the OLIF and PLIF groups (WMD − 0.07; 95% CI − 0.25 to 0.11; *I*^2^ = 11%; *P* = 0.43). However, the postoperative lumbar VAS scores were lower in the OLIF than in the PLIF group (WMD − 0.43; 95% CI − 0.84 to − 0.02; *I*^2^ = 94%; *P* = 0.04), as shown in Fig. 2b.

**Fig. 3:**
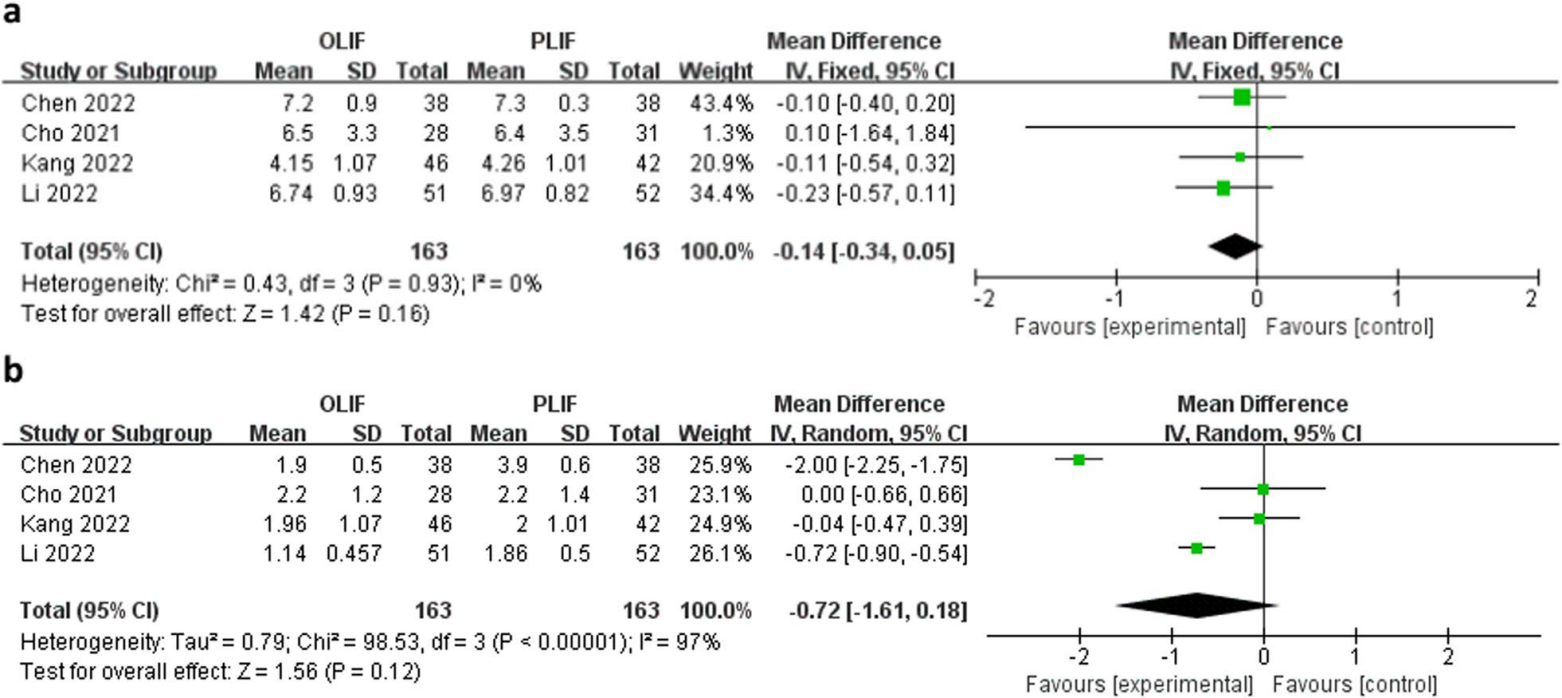
Forest plots of preoperative (**a**) and postoperative (**b**) leg visual analog scale scores between OLIF and PLIF. *CI* confidence interval, *IV* inverse variance, *df* degrees of freedom, *SD* standard deviation

Four studies assessed leg pain using VAS. There was no significant difference in the mean preoperative VAS leg pain scores (WMD − 0.14; 95% CI − 0.34 to 0.05; *I*^2^ = 0%; *P* = 0.16; Fig. 3a) and postoperative leg VAS scores (WMD − 0.72; 95% CI − 1.61 to 0.18; *I*^2^ = 97%; *P* = 0.12; Fig. 3b).

**Fig. 4:**
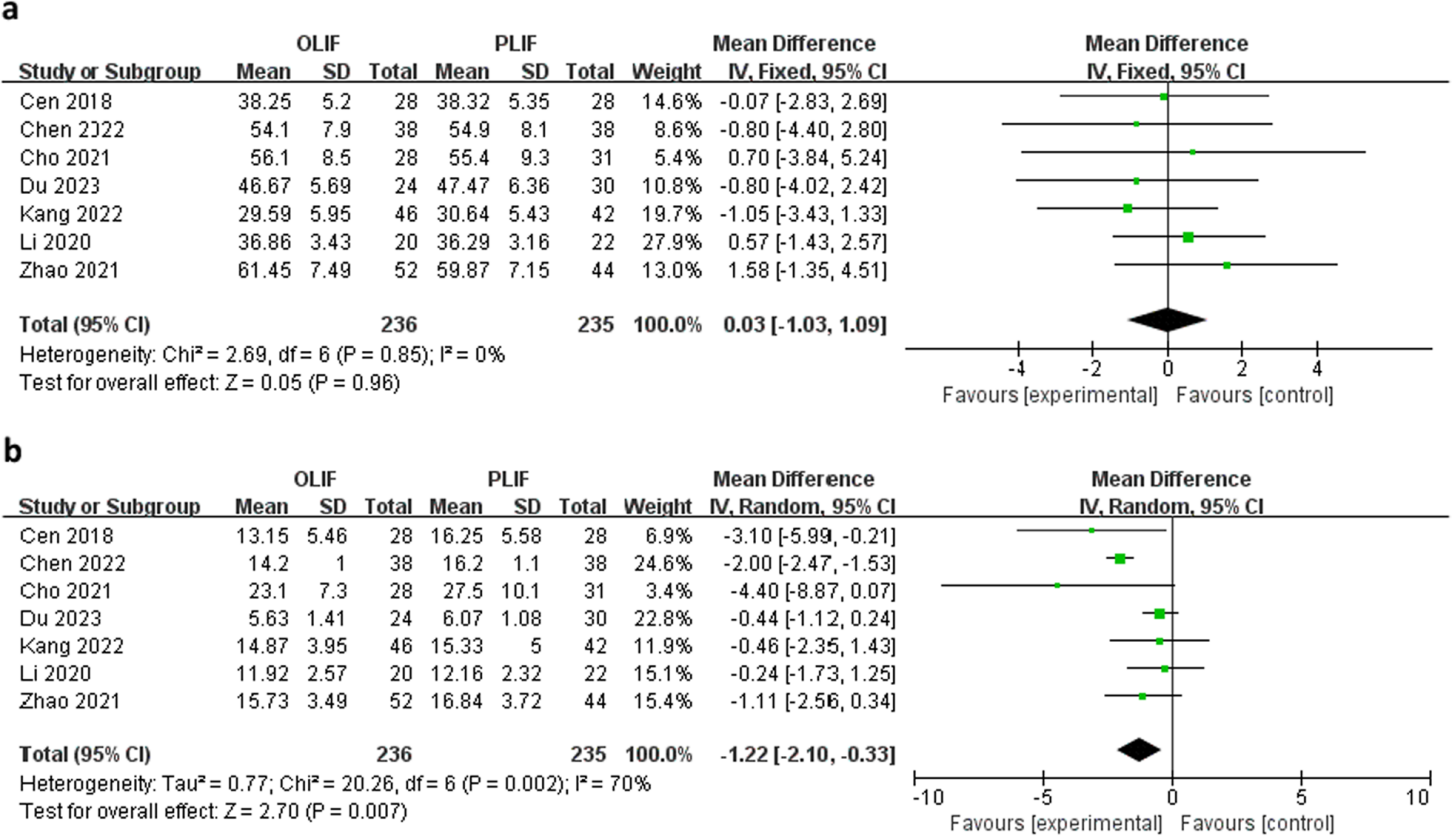
Forest plot of preoperative (**a**) and postoperative (**b**) Oswestry Disability Indexes between OLIF and PLIF. *CI* confidence interval, *IV* inverse variance, *df* degrees of freedom, *SD* standard deviation

Seven studies evaluated ODI scores. There was no difference in the mean preoperative ODI scores between the two groups (WMD 0.03; 95% CI − 1.03 to 1.09; *I*^2^ = 0%; *P* = 0.96; Fig. 4a). However, the postoperative ODI scores were significantly lower in the OLIF than in the PLIF group (WMD − 1.22; 95% CI − 2.10 to − 0.33; *I*^2^ = 70%; *P* = 0.007; Fig. 4b).

### Radiographic parameters

**Fig. 5:**
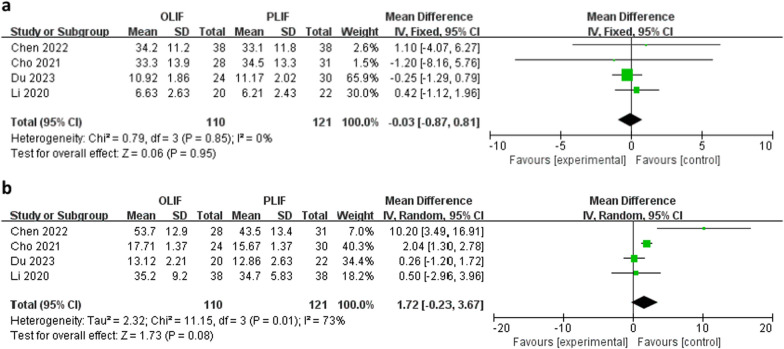
Forest plots of preoperative (**a**) and postoperative (**b**) lumbar lordosis angle between OLIF and PLIF. *CI* confidence interval, *IV* inverse variance, *df* degrees of freedom, *sd* standard deviation

Four studies evaluated lumbar lordosis angle. There was no between-group difference in the mean preoperative lumbar lordosis angle (WMD − 0.03; 95% CI − 0.87 to 0.81; *I*^2^ = 0%; *P* = 0.95; Fig. 5a) and postoperative lumbar lordosis angle (WMD 1.72; 95% CI − 0.23 to 3.67; *I*^2^ = 73%; *P* = 0.08; Fig. 5b).

**Fig. 6:**
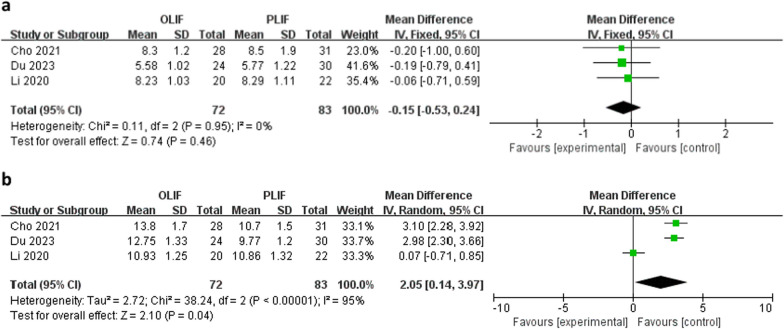
Forest plots of preoperative (**a**) and postoperative (**b**) disc heights between OLIF and PLIF. *CI* confidence interval, *IV* inverse variance, *df* degrees of freedom, *SD* standard deviation

Three studies reported no difference in mean preoperative disc height between the OLIF and PLIF groups (WMD − 0.15; 95% CI − 0.53 to 0.24; *I*^2^ = 0%; *P* = 0.46), as shown in Fig. 6a. However, as shown in Fig. 6b, postoperative disc height was higher in the OLIF than in the PLIF group (WMD 2.05; 95% CI 0.14–3.97; *I*^2^ = 95%; *P* = 0.04).

**Fig. 7:**
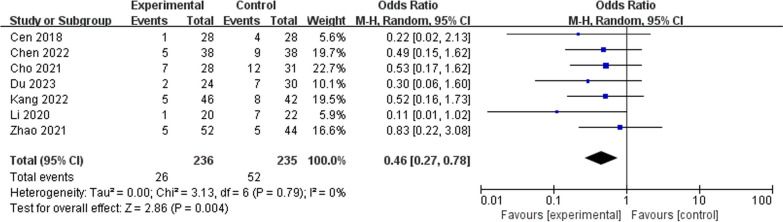
Forest plot of postoperative complication outcomes between OLIF and PLIF. *CI* confidence interval, *M–H* Mantel–Haenszel, *df* degrees of freedom, *SD* standard deviation

Seven studies compared the postoperative complications of OLIF and PLIF and reported no statistical heterogeneity in the variables (*I*^2^ = 0%, *P* = 0.79). As shown in Fig. 7, the postoperative complication rates were significantly lower in the OLIF than in the PLIF group (OR = 0.46; 95% CI 0.27–0.78; *I*^2^ = 0%; *P* = 0.004).

### Perioperative parameters

**Fig. 8:**
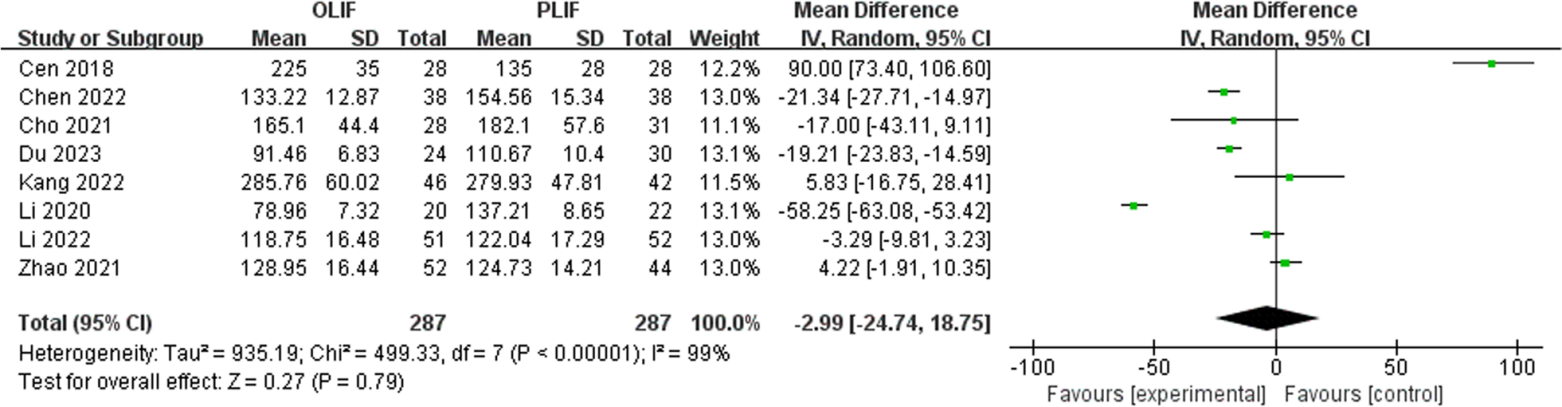
Forest plot of surgery time between OLIF and PLIF. *CI* confidence interval, *IV* inverse variance, *df* degrees of freedom, *sd* standard deviation

Eight studies reported no difference in operative time between the OLIF and PLIF groups. As shown in Fig. 8, operative time was shorter in the OLIF than in the PLIF group (WMD − 2.99; 95% CI − 24.74 to 18.75; *I*^2^ = 99%; *P* = 0.79).

**Fig. 9:**
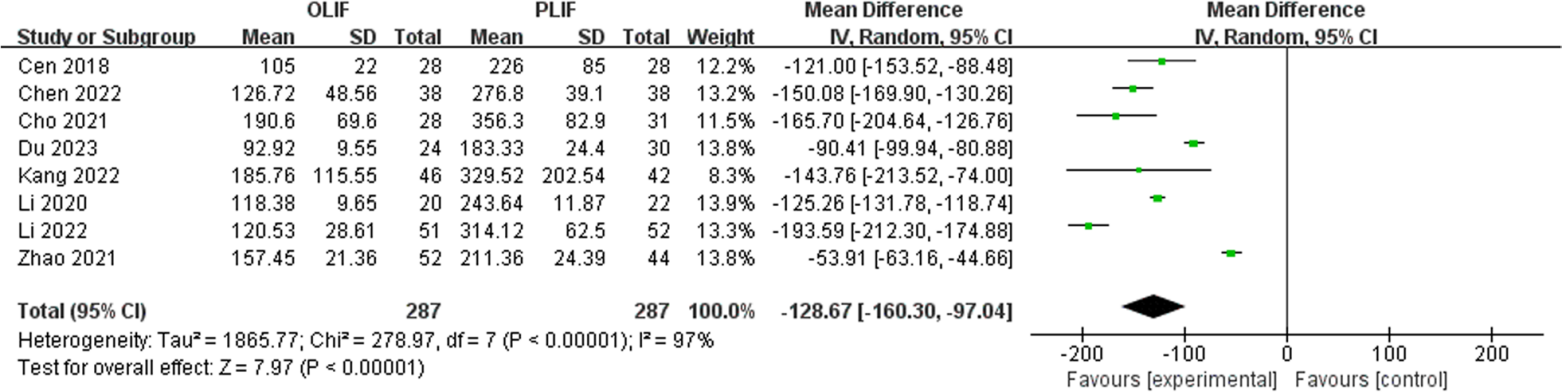
Forest plot of intraoperative blood loss between OLIF and PLIF. *CI* confidence interval, *df* degrees of freedom, *IV* inverse variance, *sd* standard deviation. Four studies reported on the length of hospital stay

**Fig. 10:**
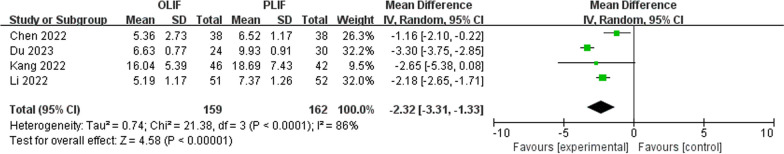
Forest plot of length of stay between OLIF and PLIF. *CI* confidence interval, *IV* inverse variance, *df* degrees of freedom, *sd* standard deviation

Intraoperative blood loss was reported in all studies. There was statistical heterogeneity in the intraoperative blood loss between the two groups (*I*^2^ = 97%, *P* < 0.00001). Intraoperative blood loss was significantly lower in the OLIF than in the PLIF group (WMD − 128.67; 95% CI − 160.30 to − 97.04; *I*^2^ = 97%; *P* < 0.00001), as shown in Fig. 9. There was also statistical heterogeneity in the length of stay between the two groups (*I*^2^ = 86%, *P* < 0.0001). As shown in Fig. 10, the length of stay was shorter in the OLIF than in the PLIF group (WMD − 2.32; 95% CI − 3.31 to − 1.33; *I*^2^ = 86%; *P* < 0.00001).

## Discussion

In this systematic review and meta-analysis, we compared the effectiveness of OLIF and PLIF in reducing disability and pain in patients with LDDs and evaluated the differences in operative time, intraoperative bleeding, length of hospital stay, and postoperative complications between the two techniques. We found no differences in preoperative VAS, ODI between patients in the two surgical groups [21–32].

However, the lumbar VAS and ODI scores were significantly lower at postoperative follow-up in the OLIF group than in the PLIF group (except for the lower extremity pain VAS score). According to Ohtori et al. [21], patients receiving OLIF showed better clinical improvement in ODI and VAS lower back pain scores in the early postoperative period than those receiving PLIF, and this may be attributed to the minimally invasive nature of OLIF. Postoperative lower back pain was associated with muscle atrophy and degeneration, emphasizing the need to minimize muscle damage during surgery [22]. Therefore, the superiority of OLIF over PLIF in terms of postoperative scores may be attributed to the absence of back muscle injury in OLIF, avoidance of direct intraoperative traction on nerve roots, and preservation of postoperative anatomical structures, including the articular surfaces, laminae, paravertebral muscles, and ligamentous structures [29, 33].

Our meta-analysis also revealed less intraoperative bleeding in the OLIF group than in the PLIF group, and this is consistent with the findings of previous studies [29–32]. According to Silvestre et al. [34], OLIF was associated with minimal blood loss and short operative time, and this may be attributed to the smaller incision used in OLIF, reducing the risk of injury to the surrounding tissues and the subsequent intraoperative bleeding [35]. In addition, the access system of OLIF is placed in the muscle gap after entering the peritoneum, thereby avoiding stripping of the soft tissues around the spine and causing less disturbance to the spinal canal; these can avoid the formation of scar tissue [31].

Furthermore, analysis of the hospitalization data showed that the length of stay was significantly shorter in the OLIF than in the PLIF group. This may be because PLIF requires longer surgical incision; extensive stripping of the muscle tissue from spinal structures, such as the spinous process, laminae, and small joints, which causes more trauma to the posterior spinal column; longer operative time; and severe intraoperative bleeding [36, 37]. These events overall result in a prolonged postoperative recovery period, implying better recovery for patients undergoing OLIF and suggesting that OLIF can potentially reduce the consumption of clinical resources and treatment costs [38].

Moreover, postoperative complication rates were higher in the PLIF than in the OLIF group (OR = 0.46), and patients receiving PLIF more often had serious complications such as Iatrogenic nerve root injury compared to those receiving OLIF. Although the overall postoperative complication rate was lower in the OLIF group, this group had a higher risk of specific complications such as retrograde ejaculation and abdominal aortic injury compared to the PLIF group [34, 39]. Therefore, some scholars question the safety of OLIF, speculating that avoiding large vessels during operation will increase the risk of massive bleeding during operation and affect the recovery of patients [31, 40]. In a meta-analysis of OLIF-related complications, transient psoas paresis was the most common complication [41]. However, given the limited scope of the relevant published literature and the associated small sample sizes, statistical analyses of the high and low incidence rates of each complication were not performed in this study. Overall, regardless of the type of complication, we observed a lower rate of complications in OLIF compared to PLIF.

This meta-analysis had some limitations. First, studies that were not RCTs (the included studies were all of retrospective design) or those assessing long-term outcomes were not included. Second, subgroup analysis of factors contributing to heterogeneity was not performed. Third, determination of disease indicators across the studies by clinicians may have involved variance, thus contributing to the potential heterogeneity of the study results. Lastly, although our selection process was thorough, the resulting sample size was small, as only a few eligible studies directly compared the metrics of OLIF and PLIF.

## Conclusions

To our knowledge, this is the first systematic review and meta-analysis to compare OLIF and PLIF. Although evidence was limited and all included studies were of retrospective design, some suggestions can be made based on our results. Both OLIF and PLIF can achieve good clinical outcomes in the treatment of LDDs; however, OLIF had higher surgical safety than PLIF in terms of intraoperative bleeding. In addition, OLIF was a superior surgical method in terms of the length of hospital stay, degree of postoperative disc height recovery, and postoperative complication rates. With the increasing demand for spine surgery, future RCTs comparing the clinical outcomes, complication rates, and cost-effectiveness between OLIF and PLIF in patients with LDDs are required to provide clinicians with a better understanding of the advantages of OLIF. Documenting long-term outcomes of patients with LDDs via publication of high-quality data will also aid in providing reliable evidence-based recommendations for clinical practice.

## Data Availability

All data generated and analyzed during this review are included in this published article and its supplementary information files.

## Abbreviations

CIs: Confidence intervals
LDDs: Lumbar degenerative diseases
DLS: Degenerative lumbar spondylolisthesis
DLSS: Degenerative lumbar spinal stenosis
MDs: Mean differences
ODI: Oswestry disability index
OLIF: Oblique lumbar interbody fusion
OR: Odds ratio
PLIF: Posterior lumbar interbody fusion
PRISMA: Preferred reporting items for systematic evaluations and meta-analyses
VAS: Visual analog scale
WMDs: Weighted mean differences

## References

[1] Kim HS, Wu PH, Jang IT (2020). Lumbar degenerative disease part 1: anatomy and pathophysiology of intervertebral discogenic pain and radiofrequency ablation of basivertebral and sinuvertebral nerve treatment for chronic discogenic back pain: a prospective case series and review of literature. Int J Mol Sci.

[2] Aoki Y, Takahashi H, Nakajima A, Kubota G, Watanabe A, Nakajima T (2020). Prevalence of lumbar spondylolysis and spondylolisthesis in patients with degenerative spinal disease. Sci Rep.

[3] Bisson EF, Guan J, Bydon M, Alvi MA, Goyal A, Glassman SD (2021). Patient-reported outcome improvements at 24-month follow-up after fusion added to decompression for grade I degenerative lumbar spondylolisthesis: a multicenter study using the quality outcomes database. J Neurosurg Spine.

[4] Sutovsky J, Sutovska M, Kocmalova M, Kazimierova I, Pappova L, Benco M (2019). Degenerative lumbar spondylolisthesis: biochemical aspects and evaluation of stabilization surgery extent in terms of adjacent segment disease theory. World Neurosurg.

[5] Cloward RB (1953). The treatment of ruptured lumbar intervertebral discs by vertebral body fusion. I. Indications, operative technique, after care. J Neurosurg.

[6] Tan Y, Tanaka M, Sonawane S, Uotani K, Oda Y, Fujiwara Y (2021). Comparison of simultaneous single-position oblique lumbar interbody fusion and percutaneous pedicle screw fixation with posterior lumbar interbody fusion using o-arm navigated technique for lumbar degenerative diseases. J Clin Med.

[7] Davis TT, Hynes RA, Fung DA, Spann SW, MacMillan M, Kwon B (2014). Retroperitoneal oblique corridor to the L2–S1 intervertebral discs in the lateral position: an anatomic study. J Neurosurg Spine.

[8] Mobbs RJ, Phan K, Malham G, Seex K, Rao PJ (2015). Lumbar interbody fusion: techniques, indications and comparison of interbody fusion options including PLIF, TLIF, MI-TLIF, OLIF/ATP, LLIF and ALIF. J Spine Surg.

[9] Shimizu T, Fujibayashi S, Otsuki B, Murata K, Matsuda S (2021). Indirect decompression via oblique lateral interbody fusion for severe degenerative lumbar spinal stenosis: a comparative study with direct decompression transforaminal/posterior lumbar interbody fusion. Spine J.

[10] Mayer HM (1997). A new microsurgical technique for minimally invasive anterior lumbar interbody fusion. Spine.

[11] Li R (2022). Efficacy of oblique lateral lumbar interbody fusion in the treatment of degenerative lumbar spinal stenosis and its effect on oxidative stress index. Chin J Clin Anat.

[12] Cho MS, Seo EM (2021). Efficacy and radiographic analysis of oblique lumbar interbody fusion in treating lumbar degenerative spondylolisthesis with sagittal imbalance. Neurosurg Rev.

[13] Liu J, Feng H (2020). Oblique lateral interbody fusion (OLIF) with supplemental anterolateral screw and rod instrumentation: a preliminary clinical study. World Neurosurg.

[14] Xu DS, Walker CT, Godzik J, Turner JD, Smith W, Uribe JS (2018). Minimally invasive anterior, lateral, and oblique lumbar interbody fusion: a literature review. Ann Transl Med.

[15] Moher D, Liberati A, Tetzlaff J, Altman DG, PRISMA Group (2009). Preferred reporting items for systematic reviews and meta-analyses: the PRISMA statement. BMJ.

[16] Shafshak TS, Elnemr R (2021). The visual analogue scale versus numerical rating scale in measuring pain severity and predicting disability in low back pain. J Clin Rheumatol.

[17] Fairbank JC, Pynsent PB (2000). The Oswestry disability index. Spine.

[18] Stang A (2010). Critical evaluation of the Newcastle–Ottawa scale for the assessment of the quality of nonrandomized studies in meta-analyses. Eur J Epidemiol.

[19] Been E, Kalichman L (2014). Lumbar lordosis. Spine J.

[20] Zheng J, Shen C (2022). Quantitative relationship between the degree of lumbar disc degeneration and intervertebral disc height in patients with low back pain. Contrast Media Mol Imaging.

[21] Ohtori S, Mannoji C, Orita S, Yamauchi K, Eguchi Y, Ochiai N (2015). Mini-open anterior retroperitoneal lumbar interbody fusion: oblique lateral interbody fusion for degenerated lumbar spinal kyphoscoliosis. Asian Spine J.

[22] Kou Y, Chang J, Guan X, Chang Q, Feng H (2021). Endoscopic lumbar interbody fusion and minimally invasive transforaminal lumbar interbody fusion for the treatment of lumbar degenerative diseases: a systematic review and meta-analysis. World Neurosurg.

[23] Li JP, Wang QF (2020). Comparison of OLIF and PLIF in the treatment of degenerative lumbar spondylolisthesis. Chin for Med res.

[24] Cen BW (2018). Analysis of the efficacy of minimally invasive expandable channel-assisted OLIF approach in the treatment of single-segment lumbar instability. Chin J Mod Operative Surg.

[25] Chen Y (2022). Comparison of oblique lateral lumbar interbody fusion and posterior lumbar interbody fusion in the treatment of scoliosis. Chin J Bone Joint Inj.

[26] Zhao FY (2021). Clinical efficacy of posterior lumbar interbody fusion versus oblique posterolateral lumbar interbody fusion for the treatment of degenerative lumbar spine disease. Bio-Orthop Mater Clin Res.

[27] Du W, Wang Z, Dong Y, Hu J, Quan R, Qi J (2023). Recent efficacy of oblique lateral interbody fusion combined with Wiltse approach pedicle screw fixation for degenerative single-level lumbar spondylolisthesis. World Neurosurg.

[28] Kang GH, Son D, Lee JS, Lee SH, Bae SH, Lee SW (2022). Surgical treatment for degenerative lumbar disease with neurologic deficits: comparison between oblique lumbar interbody fusion and posterior lumbar interbody fusion. Korean J Neurotrauma.

[29] Zhu G, Hao Y, Yu L, Cai Y, Yang X (2018). Comparing stand-alone oblique lumbar interbody fusion with posterior lumbar interbody fusion for revision of rostral adjacent segment disease: a STROBE-compliant study. Medicine.

[30] Yang Z, Chang J, Sun L, Chen CM, Feng H (2020). Comparing oblique lumbar interbody fusion with lateral screw fixation and transforaminal full-endoscopic lumbar discectomy (OLIF-TELD) and posterior lumbar interbody fusion (PLIF) for the treatment of adjacent segment disease. BioMed Res Int.

[31] Jin C, Xie M, He L, Xu W, Han W, Liang W (2019). Oblique lumbar interbody fusion for adjacent segment disease after posterior lumbar fusion: a case-controlled study. J Orthop Surg Res.

[32] Wu M, Li J, Zhang M, Ding X, Qi D, Li G (2019). Efficacy and radiographic analysis of oblique lumbar interbody fusion for degenerative lumbar spondylolisthesis. J Orthop Surg Res.

[33] Lin GX, Akbary K, Kotheeranurak V, Quillo-Olvera J, Jo HJ, Yang XW (2018). Clinical and radiologic outcomes of direct versus indirect decompression with lumbar interbody fusion: a matched-pair comparison analysis. World Neurosurg.

[34] Silvestre C, Mac-Thiong JM, Hilmi R, Roussouly P (2012). Complications and morbidities of mini-open anterior retroperitoneal lumbar interbody fusion: oblique lumbar interbody fusion in 179 patients. Asian Spine J.

[35] Abe K, Orita S, Mannoji C, Motegi H, Aramomi M, Ishikawa T (2017). Perioperative complications in 155 patients who underwent oblique lateral interbody fusion surgery: perspectives and indications from a retrospective, multicenter survey. Spine.

[36] Chen WJ, Lai PL, Niu CC, Chen LH, Fu TS, Wong CB (2001). Surgical treatment of adjacent instability after lumbar spine fusion. Spine.

[37] Freudenberger C, Lindley EM, Beard DW, Reckling WC, Williams A, Burger EL (2009). Posterior versus anterior lumbar interbody fusion with anterior tension band plating: retrospective analysis. Orthopedics.

[38] Nohara A, Kawakami N, Saito T, Tsuji T, Ohara T, Suzuki Y (2015). Comparison of surgical outcomes between anterior fusion and posterior fusion in patients with AIS Lenke type 1 or 2 that underwent selective thoracic fusion -long-term follow-up study longer than 10 postoperative years. Spine.

[39] Berjano P, Balsano M, Buric J, Petruzzi M, Lamartina C (2012). Direct lateral access lumbar and thoracolumbar fusion: preliminary results. Eur Spine J.

[40] Li JX, Phan K, Mobbs R (2017). Oblique lumbar interbody fusion: technical aspects, operative outcomes, and complications. World Neurosurg.

[41] Li HM, Zhang RJ, Shen CL (2019). Differences in radiographic and clinical outcomes of oblique lateral interbody fusion and lateral lumbar interbody fusion for degenerative lumbar disease: a meta-analysis. BMC Musculoskelet Disord.

